# NAL-NL2 Prescriptive Targets for Bone Conduction Devices With an Adaptation to Device Constraints in the Low Frequencies

**DOI:** 10.1097/AUD.0000000000001235

**Published:** 2022-05-30

**Authors:** Martijn Toll, Gertjan Dingemanse

**Affiliations:** 1 Department of Otorhinolaryngology and Head and Neck Surgery, Erasmus MC, University Medical Center Rotterdam, The Netherlands.

**Keywords:** Bone conduction, Bone conduction device, Bone conduction hearing, Force levels, Hearing aid, Hearing aids, Prescription, Validation, Verification

## Abstract

To study the effectivity of a transformed NAL non-linear version 2 (NAL-NL2) gain prescription for percutaneous bone conduction devices (BCDs) and to investigate how to take into account output constraints for the lower frequencies.

**Design:**

The NAL-NL2 prescription was converted to a bone conduction prescription rule. Adaptations were needed, as this converted rule prescribes more output at low frequencies than the device delivers. Three adaptations with different audibility and compression were compared. Setting 1 (S1, “optimal audibility”) had most audibility due to adapted frequency-dependent compression, setting 2 (S2, “moderate audibility”) had moderate output reduction below 1 kHz, and setting 3 (S3, “reduced audibility, least distortion”) had most output reduction. Eighteen experienced BCD users rated their relative sound quality in paired comparisons for different sounds (own voice, mixed voices, traffic noise, and music). In addition speech intelligibility in quiet and noise were assessed.

**Results:**

The relative sound quality rating for the adapted prescriptions varied between the stimuli: more low-frequency sound was preferred for music (S1 over S3), and less low-frequency sound was preferred for the own voice (S2 and S3 over S1). No differences in quality rating were found for mixed voices or traffic noise. Speech intelligibility in quiet scores at 45 dB SPL was significantly lower for S3 than for S1. Speech intelligibility in noise was significantly reduced in all settings and S3 yielded significantly better speech intelligibility in noise than S1.

**Conclusions:**

With a moderate gain reduction for low frequencies to comply with device constraints the transformed NAL-NL2 prescription was found suitable for fitting BCDs. Perceived sound quality depended on the gain settings, but also on the sound spectra and how the sound was appreciated. A moderate gain reduction below 1 kHz seems to be the optimal adaptation as it has a neutral or positive relative sound quality for all stimuli without negative effects on Speech intelligibility. The NAL-NL2-BC prescribed a sufficient amount of gain, as indicated by the speech tests.

## INTRODUCTION

Bone conduction devices (BCDs) are a well-established treatment for persons with a conductive or mixed hearing loss for whom conventional hearing aids are not an option, for example in case of ear canal atresia or chronically draining ears (Snik et al. 2005; Gavilan et al. 2015; van Barneveld et al. 2018). A BCD converts sound to a (local) vibration of the skull. Commonly, a BCD is applied percutaneously, that is, a sound processor is coupled to a surgically implanted titanium screw in the mastoid. This application provides better results than a passive transcutaneous application of BCDs (without penetration through the skin), as the skull vibration generated by the sound processor stimulates the cochlea most directly, without attenuation due to the skin (Håkansson et al. 1984; Christensen et al. 2010; Snik 2018).

The force needed to obtain sufficient BC output for audibility depends on the impedance of the skull, which is highest for the lower frequencies (Stenfelt and Goode 2005). This is reflected by the reference equivalent threshold force level (RETFL), that is, the force needed to stimulate at the threshold for normal hearing per frequency, that is relatively high a low frequencies (Carlsson et al. 1995; Flynn and Hillbratt 2012). BCD’s need to exert these forces to deliver audible sounds for normal cochlear thresholds.

The dynamic range of a BCD for a patient is defined by his/her hearing threshold (e.g., the cochlear loss plus the RETFL) and the maximum force output (MFO), the loudest sounds that can be delivered by the BCD. This MFO decreases below the resonance frequency of the device, which is usually 750 to 1000 Hz. The consequence of high RETFLs and a limited MFO is a limited dynamic range, especially in the lower frequencies. This limited dynamic range in its turn limits the fitting range, i.e. the maximum sensorineural hearing loss that can be adequately compensated with a specific BCD. This limitation should be taken into account when prescribing the required amplification in case of a sensorineural component in a hearing loss.

To assist the clinician in fitting BCDs, manufacturers offer proprietary fitting procedures. A Quality standard for bone conduction implants (Gavilan et al. 2015) recommends that “each device should be fitted and programmed according to the manufacturer’s recommended procedures to maximize benefit for the patient.” However, given the lack of published evidence for manufacturer fitting procedures, it is not clear how the proprietary fitting procedures are developed and take the limited dynamic range into account. Some researchers mention the limited dynamic range and stated that a phoneme perception score in quiet of at least 50% at 65 dB SPL must be achieved with a BCD (Snik et al. 2005) or advised a dynamic range of 35 dB at minimum (Zwartenkot et al. 2014). This seems a little ambitious for pure conductive or mixed hearing losses with only a mild sensorineural component. A recent survey on fitting BCDs in children (Gordey and Bagatto 2021) confirms this lack of evidence-based fitting rules for children and concluded that clinicians lack a feeling of confidence while fitting BCDs in children. They suggested the development of standardized fitting practices with suitable prescriptive targets and appropriate verification tools.

For air conduction (AC) hearing aids several evidence-based prescription rules for output and gain are available. The desired sensation level (DSL) prescription for instance aims to provide an audible and comfortable signal in each frequency band and maximized bandwidth (Scollie et al. 2005), whereas the prescriptions of the NAL aim to optimize speech intelligibility at listening level based on effective audibility for all contributing frequency bands and comfortable overall loudness (Keidser et al. 2011; Dillon 2012). The most recent version of the DSL (DSL-v5) and the most recent NAL prescription (NAL non-linear version 2 [NAL-NL2]) provide on average comparable overall loudness and predicted speech intelligibility in quiet and noise, although differences in gain prescription over frequencies exist between both fitting rules, depending on the audiogram configuration (Johnson and Keidser 2011). To verify whether the prescribed gain or output is achieved, real ear measurements (REM), measuring the sound pressure level at the eardrum (ED) with the AC hearing aids in situ, are the gold standard.

In 2017 (Hodgetts and Scollie 2017) proposed a BCD fitting prescription based on the fifth version of the DSL (DSL v5.0) prescription for AC hearing aids. This DSL-BCD fitting rule uses the BC thresholds as input. The DSL prescribed output in dB SPL is transformed to prescribed output of a BCD in force level (FL). The DSL aims to optimally map the incoming sound into the available limited dynamic range. As (Hodgetts and Scollie 2017) report, the upper limit of the fitting of a BCD can be either the uncomfortable loudness levels for bone conduction stimulation (a patient characteristic) or the MFO of the BCD (a device characteristic).

To verify whether the BCD output matches the prescribed output force, a skull simulator is used. A skull simulator is an accelerometer with a known mechanical impedance that simulates the mechanical impedance of the human skull. Skull simulator measurements are a good verification option for percutaneous coupled BCDs, analog to the Hearing Instrument Test or REM for AC hearing aids (Hodgetts et al. 2011), where one should realize the REM reflects personal aspects where both the Hearing Instrument Test and the skull simulator measurements do not.

During the development of the DSL-BCD, some empirical adjustments were made after the transformation of the DSL targets for AC hearing aids to targets for BCD, mainly because participants reported a lower sound quality and a barrel effect for their own voice. To facilitate acceptation of own voice, the low-frequency targets were lowered. These adjustments were practice-based, and no systematic approach was used.

In this study, we transformed the NAL-NL2 prescription of output in dB SPL for AC hearing aids to output in dB FL for BCD. The NAL-NL2 fitting rule, has a proven rationale based on the effective contribution of each frequency band to good speech intelligibility, while keeping risk on uncomfortable loudness low (Johnson 2013). The NAL-NL2 takes the hearing loss desensitization into account, that is, the decrease of intelligible speech information that can be extracted from an audible signal as hearing loss increases (Ching et al. 2011; Keidser et al. 2011). This factor reduces the prescribed gain mainly for larger hearing losses that are not common in BCD fittings. But in cases of higher bone conduction thresholds for the high frequencies, the NAL-NL2 prescribed gain may be reduced by the desensitization factor, which may be an advantage in application of the NAL-NL2 in BCDs that have limited maximum output and gain.

The NAL-NL2 prescription of gain for BCDs is based on the bone conduction thresholds reflecting the sensorineural part of the hearing loss. As the BCD stimulates the cochlea by skull vibration, the air-bone gap is bypassed.

This NAL-NL2-BC prescription needs to be adapted to comply with the limited dynamic range due to device constraints, especially in the lower frequencies. For the frequencies below 1 kHz, the dynamic range is limited most, and therefore the difference between transformed prescription and realizable output is biggest. In addition, low-frequency amplification is related to several potential problems specific for BCD.

First, the low-frequency region is associated with reported problems such as insufficient quality of the own voice (Snik et al. 2005; Snik 2015; Hodgetts and Scollie 2017).

Second, signal processing in modern hearing devices include multichannel fast compression limiting that prevents peak clipping if the MFO in a frequency band is reached (McDermott et al. 1999). Limiting compression uses fast attack times and larger compression ratios, and results in some signal distortion (Stone and Moore 2007). In BCDs, the MFO is often reached in the lower frequencies, even at normal speech levels. Listeners may perceive some saturation distortion and changes in timbre. A lower gain to avoid such signal alterations may result in better-perceived sound quality.

Third, in BCDs, the sound quality may also be influenced by harmonic distortion that occurs mainly at frequencies below 1 kHz (roughly the resonance frequency) and for higher stimulation levels, most at 250 Hz (Arlinger et al. 1978; Eichenauer et al. 2014). Harmonic distortion spreads the energy of the signal to higher frequencies and this results in an increase in loudness (Stenfelt and Håkansson 2002) and possibly some perceivable disturbance. There is no specific criterion for when harmonic distortion becomes disturbing, but more than 10% is regarded as too much (Agnew 1998).

This study intended to investigate the application of a NAL-NL2–based fitting rule for BCDs. Given the limited dynamic range for lower frequencies along with possible side effects of distortion in the lower frequencies, an adaptation to these device constraints is necessary.

The objective of this study was to answer the questions:

1. What is the optimal adaptation to low-frequency device limitations in terms of sound quality and speech intelligibility in quiet and noise?
2. What is the effectivity of the adapted NAL-NL2 BC fitting rule as measured with speech intelligibility in quiet and noise?

We compared three different adaptations of the NAL-NL2-BC by asking study participants to rate sound quality for different sounds. As the perception of the quality of the own voice may be important, a judgment of the quality of the own voice was included. We hypothesize that sound quality is optimal if the low-frequency output contributes to the sound perception (Hua et al. 2021) but at the same time is well below the MFO to avoid distortion.

In addition, we assessed speech intelligibility in noise and quiet to test the influence of the adaptations on this speech intelligibility. The hypothesis is that a reduced output for frequencies below 1 kHz has little or no effect on speech intelligibility in quiet and noise for normal speech intensities. The reduction is largest below 500 Hz and the contribution of this frequency region to speech intelligibility is relatively small according to the Speech Intelligibility Index (ANSI 1997).

## MATERIALS AND METHODS

### Participants

**Fig. 1.**
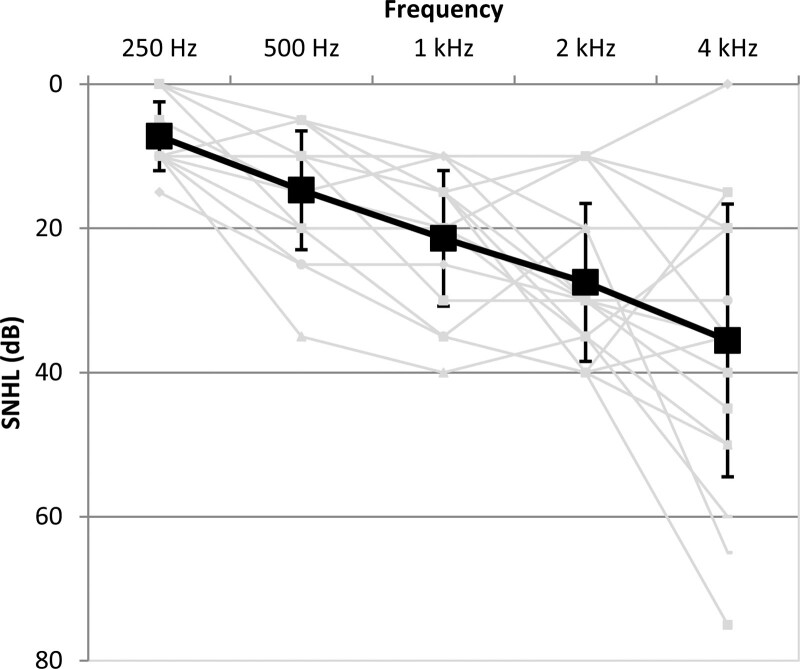
BC in-situ thresholds for the test ear. In black mean thresholds with error bars based on SD, in gray individual thresholds.

Eighteen BCD users, nine female and nine male, with conductive or mixed hearing loss participated in this study. They had near-normal cochlear function to slight sensorineural component in the hearing loss for the test ear. The mean pure tone average for 250, 500, and 1000 Hz was 14 dB HL for BC in-situ thresholds (i.e. the BC thresholds measured with the BCD in situ). Figure 1 shows the average and individual BCD in-situ thresholds. The mean AC pure tone average for 250, 500, and 1000 Hz was 55 dB HL for the test ear and 72 dB HL for the contralateral side. The mean pure tone average for 250, 500, and 1000 Hz for BC thresholds was 48 dB HL for the non-test ear. Contralateral speech discrimination at normal speech levels was negligible, that is, less than 30%. Participants used their BCD for at least 6 months before the test and the basis of their BCD fitting was the gain prescription of the manufacturer, with for some participants extra amplification for audibility and fine-tuning based on their feedback. Participants ranged in age from 16 to 77 years (group mean 59 years; SD = 16) and were all native Dutch speakers. For inclusion in this study, an aided phoneme score of at least 70% in quiet on clinically used Dutch consonant–vowel–consonant word lists (Bosman and Smoorenburg 1995) was required and virtually no unaided speech perception at 65 dB SPL on the contralateral side. Participants signed a written informed consent form before participating in the study. Approval of the study protocol was obtained by the Ethics Committee of the Erasmus Medical Center.

### BCD Output Prescription

To obtain a NAL-NL2-based fitting for the BCD, we transformed the NAL-NL2 gain prescription for AC to a NAL-NL2-BC output in FL. The input of the NAL-NL2 gain calculations was the sensorineural part of the hearing loss. The result of this transformation was a prescribed output that exceeded the MFO of the test device and hence had to be adapted to that device.

### NAL-NL2 Transformation to Force Level

The transformation from an AC gain (G_NAL-aC)_ to BC output (O_NAL-BC_) consisted of four steps:

1. Measurement of in-situ hearing thresholds in dB HL per participant. With a skull simulator measurement, we confirmed per octave frequency that 0 dB HL in-situ stimulation corresponded to the RETFL (Carlsson et al. 1995) within 3 dB.
2. Based on these thresholds, used as if they were AC-thresholds, the NAL-NL2 prescribed insertion gain (G_NAL-AC_) was derived for input levels of 55, 65, and 75 dB SPL with parameters “binaural,” “experienced user,” and “non-tonal.” For the “gender” parameter, the gender of the participants was used.
3. The prescribed gain was transformed to output at ED (O_ED-AC_), with the International Long-term Average Speech Spectrum (ILTASS) of a female voice (Byrne et al. 1994), using O_ED-AC_ = G_NAL-AC_ + ILTASS +T_FF2ED_, where G_NAL-AC_ is the gain prescribed by the NAl-NL2 and T_FF2ED_ is the transformation from intensity in free field (FF) to intensity at ED.

Microphone location effects were neglected in the transformation, as they are small (<2 dB) for frequencies below 1 kHz (Franks et al. 1981; Gartrell et al. 1990).

4. The prescribed output was transformed to output in FL, based on equal sensation levels (output minus hearing threshold) using O_NAL-BC_ = O_ED-AC_ − REDD + RETFL, where REDD is the real ear to dial difference that gives the transformation of hearing thresholds in dB audiometer level to dB SPL (Scollie et al. 1998).

### Adaptation of the Transformed NAL-NL2 Prescription

**Fig. 2.**
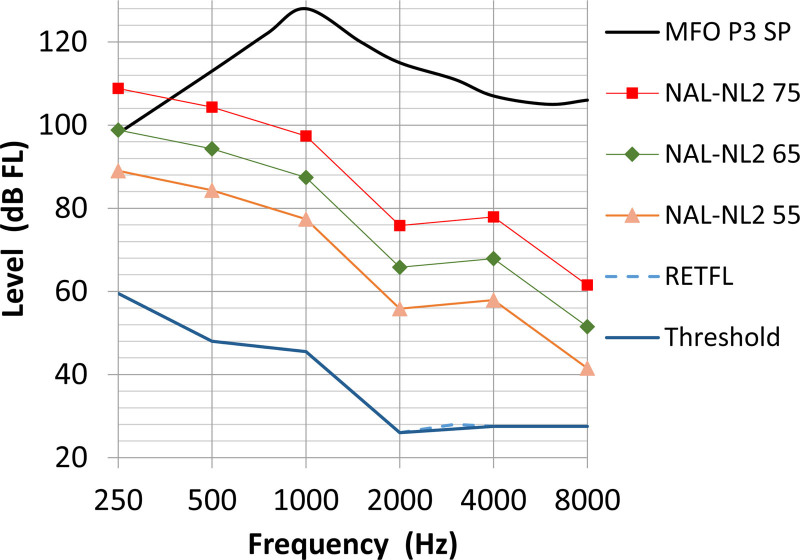
The prescribed bone conduction output OBC for three different input levels for normal cochlear thresholds (solid blue line). Red, green, and orange lines are the prescribed outputs for 55, 65, and 75 dB SPL input. The hearing thresholds are equal to the reference equivalent thresholds in force level (RETFL) (dashed blue line). The black line indicates the maximum force output of the Ponto 3 SP, the device used in this study and the region above the black line is the output that cannot be delivered by the device. dB SPL indicates decibel sound pressure level.

In this study, Ponto 3 Superpower BCDs (Oticon Medical, Askim, Sweden) were used. Figure 2 shows the transformed NAL-NL2 prescription O_NAL-BC_ for input of 55, 65, and 75 dB SPL for 0 dB hearing threshold together with the MFO of the Oticon Medical Ponto 3 SP. Where the prescription exceeds the MFO, adaptations for device constraint are needed, i.e. the amplification at these low frequencies had to be reduced. In this adaptation we used two variables: the minimum headroom *H* and the compression ratio *CR*. We define the headroom H as the difference between MFO and the output for an input of loud speech (ILTASS at 75 dB SPL [henceforth, output 75]) H = MFO − output 75. The compression ratio CR is defined as $CR=\frac{\Delta Input}{\Delta Output}$. For H we chose two different values: 12 dB as a minimum, based on the dynamic properties of speech (e.g., Rhebergen et al. 2009) and 20 dB to ensure that no saturation occurred and distortion was minimized. For the CR we also used two different values, CR = 3 for optimal audibility and normal NAL-NL2 compression ratios. The three different adaptations made are:

S1: setting 1: “optimal audibility”: H = 12, CR = 3;

S2: setting 2: “moderate audibility”: H = 12, CR = CR_NAL-NL2;_

S3: setting 3: “reduced audibility, least distortion”: H = 20, CR = CR_NAL-NL2_.

**Fig. 3.**
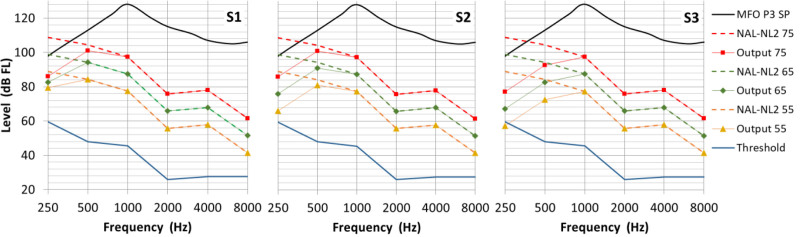
Three different adaptations, S1, S2, and S3 to the prescribed bone conduction output for normal cochlear thresholds. The prescribed bone conduction output OBC for three different input levels are indicated in dashed red, green, and orange lines. The black line indicates the maximum force output of the Ponto 3 SP, the device used in this study and blue line is the hearing threshold for normal cochlear function. Note red, green and orange lines converge in S1 for lower frequencies, indicating more compression. Prescribed output has more headroom in S3.

Results of these adaptations are shown in Figure 3, also depicted for normal cochlear function. Effects of effective stimulation and dynamic range are clearly visible. At frequencies above 1 kHz the MFO was sufficient, so no adaptation for minimum headroom was needed. Note that these changes are frequency-dependent and will change timbre.

### BCD Fitting and Verification

The output of the BCD was verified and adjusted for each adapted prescription at different input signals (ISTS at 55, 65, 75 dB SPL), using a skull simulator (SKS 10 Interacoustics, Copenhagen, Denmark) and Hearing Instrument Test module (Affinity Interacoustics, Copenhagen, Denmark). It was possible to fit to the prescribed levels within a margin of 3 dB below 1 kHz. For frequencies above 1 kHz the feedback thresholds were variable and some participants had elevated thresholds, especially at 4 and 8 kHz. High frequency output was as good as possible to conform to NAL-NL2-BC and constant over settings.

The three different prescriptions were programmed in three program slots of the BCD. The order of the three settings in the program slots was balanced across participants with a repeating 3x3 Latin Square. During the experiment, the participants used program 1, 2, and 3 in fixed order. This procedure was intended to create a double blind situation, in the sense that the experimenter and participants were not informed about the order of the fitting strategies. However, perhaps the experimenter or participant could deduce a strategy from the perceived sound, ratings or comments given by the participants. In all programs, noise reduction and directionality algorithms were switched off.

### Test Procedures

In this prospective study a within-subject repeated measures design was used. All participants underwent pure tone audiometry and speech audiometry at 65 dB SPL with and without their own BCD if no recent (<3 months) audiometry was available. BC thresholds were obtained with the test device (BC in-situ audiometry) and based on these outcomes inclusion was checked. If inclusion criteria were met, the participant and experimenter had a pause while the device was fitted by one of the authors.

After these preparations speech recognition was tested in quiet and in noise for the different test conditions. The paired comparisons were at the end of the test session. The non BCD ear was unaided during the tests and was left open and unmasked, as the inclusion criteria warranted that it not contributed to speech intelligibility. All tests with the BCD were done in FF with sounds presented from the front.

### Subjective Sound Quality Comparisons

A paired comparison approach was used to assess the perceived quality of the BCD settings relative to each other in laboratory. The three programs were compared with each of the other programs. In each comparison, participants listened to program A first, then program B, program A again and finally program B. Afterwards they were asked to rate the perceived sound quality using a seven-alternative forced choice graded response, with three ordinal difference magnitudes, ranging from “B is much better than A” to “B is much worse than A,” and an “equal” grade. The answers were transformed into numbers ranging from −3 to 3. For example, if the subject rated the sound quality of program A slightly better than program B, then a score of 1 was assigned to program A and a score of −1 to program B. The seven choice categories and the transformation to numbers were in accordance with the Comparison Category Rating method described in (ITU-P, I. T. U. 1996, annex E.1). Spontaneous comments on the settings were also noted by the examiner and analyzed for consistency.

The paired comparisons were performed with different sounds: (1) a fragment of mixed voices: speech by a male voice with a background of two female voices; (2) traffic noise at a busy street with a car accelerating; (3) a fragment of staccato violin music, and (4) the participants’ own voice when they read aloud a piece of text. During the reading, the own voice was recorded once per condition and the long-term spectrum of the second sentence per condition was derived afterwards. The order of the sound fragments was balanced across programs and participants.

**Fig. 4.**
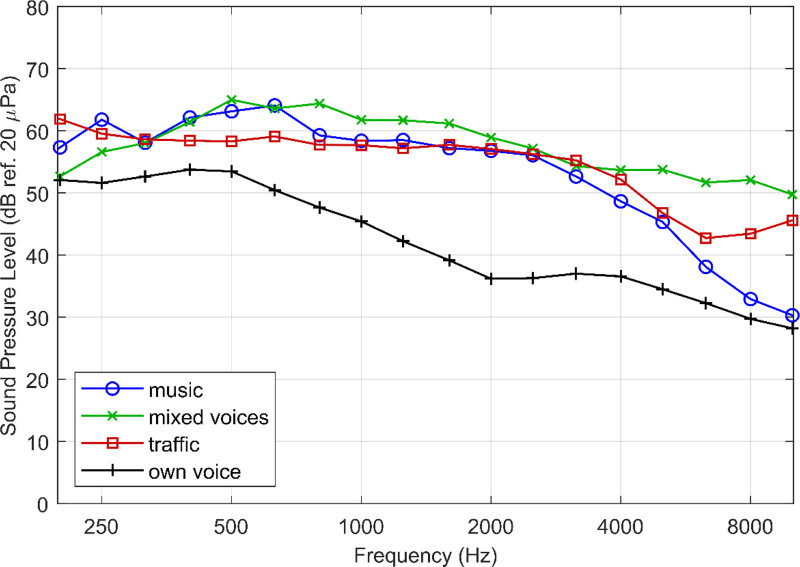
One-third octave spectra of the different sounds used in the paired comparisons, in black the recorded own voice, averaged over participants. In blue, green, and red, respectively music, mixed voices, and traffic noise.

Figure 4 shows the spectra of these sounds. The black line is the between-participants average of their own voice. The recorded own voice at the position of the ear of the participants has *on average over participants* the lowest intensity. The effective difference between the different settings comes from the frequency region between 200 and 1000 Hz, as the output of the device for the three settings is programmed the same at frequencies above 1 kHz and the output is barely above normal hearing threshold for frequencies lower than 200 Hz. As one can see the sounds do not only differ in connotation but also in frequency.

### Speech Intelligibility Tests

To test speech intelligibility in quiet, we used the standard clinically used Dutch speech test of the Dutch Society of Audiology (Bosman and Smoorenburg 1995), which consists of phonetically balanced monosyllabic CVC (consonant–vowel–consonant) word lists. The word lists were presented at 45, 55, and 65 dB SPL. At 45 dB SPL, the list was presented twice to improve accuracy.

For testing speech intelligibility in noise, female Dutch speech material developed at the VU Medical Center was used (Versfeld et al. 2000). A total of 26 sentences were presented at a fixed level of 70 dB SPL speech and noise together. This level is representative for that of a raised voice in noisy situations (Pearsons 1977). Both speech and noise came from the front (S_0_N_0_). The sentences were presented in steady-state, speech-shaped noise with a varying signal-to-noise ratio (SNR) using a stochastic approximation adaptive procedure (Dingemanse and Goedegebure 2019) to estimate the SNR that yielded a target score of 50% correctly recognized words. For this speech-in-noise test, the norm value in young persons with normal hearing is −5.5 dB SNR.

### Equipment

The experiments were done with two new Oticon Medical Ponto 3SP devices (one for left side use, one for right side use). The fitting of the BCD and the BC in-situ assessment was done in a quiet consultation room. All other tests and sound quality comparisons were performed in a sound-treated room. For FF tests, participants sat 1 m in front of a loudspeaker, Genelec 8020D, Iisalmi, Finland. The speech-in-noise test and the paired comparisons stimuli were presented in a custom application (cf. Dingemanse et al. 2018) running in Matlab R2019a (The MathWorks Inc., Natick, MA).

Harmonic distortion was measured with the SKS 10 skull simulator with the Affinity software in HIT module. The harmonic distortion was measured with pure tones of 250, 500, and 1000 Hz and 65 and 75 dB SPL input level, with the BCD fitted to the median hearing threshold values of all participants.

We recorded the voice of each participant with a microphone, type Q1U (Samson Technology, Hickville, NY). From these recordings, the overall intensity was assessed and the fundamental frequency of the voice was derived with Praat software (Boersma and David 2019).

### Data Analysis

An a priori power analysis using G*power software (Faul et al. 2007) indicated that 17 participants would be needed to yield a clinically significant difference of 10% points for speech intelligibility in quiet. Data interpretation and analysis were performed with Matlab (R2019a; The MathWorks Inc.). We assumed a non-normal distribution of the paired comparison data and analyzed it with a one-sample Wilcoxon Signed Rank test. The CVC phoneme scores were transformed into rationalized arcsine units for statistical analysis, according to (Studebaker 1985). We used paired t-tests to analyze the significance of differences in CVC scores and speech reception thresholds (SRTs). In cases of multiple comparisons, we used the Benjamini–Hochberg method to control the false discovery rate at level 0.05 (Benjamini and Hochberg 1995).

## RESULTS

### Sound Quality Comparisons

**Fig. 5.**
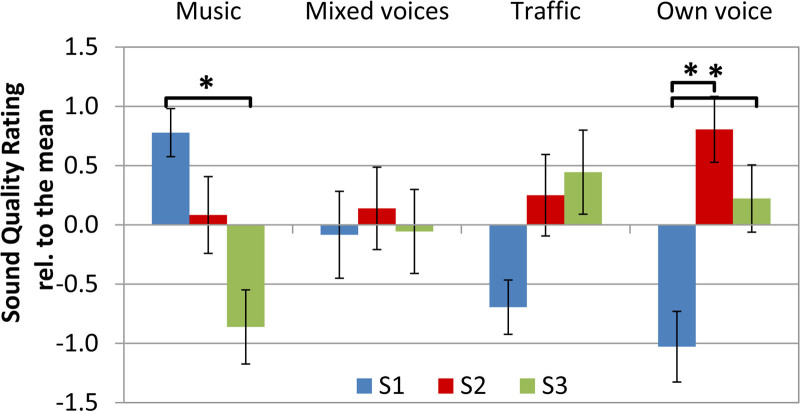
Mean sound quality ratings relative to the mean. Errorbars represent SEs. In the figure, the relative appreciation of the three different sounds that were played and the appreciation of the own voice was rated over the three different adaptation strategies S1, S2, and S3. Positive values indicate relative appreciation for a prescription strategy.

The results of the paired comparisons are presented in Figure 5 and show differences in the relative sound quality for the prescription settings between the sounds that the participants had to assess. Positive scores indicate relative appreciation. For *music*, the relative sound quality was highest for S1 (“optimal audibility”), which rating was significantly better than that of S3 (“reduced audibility, least distortion”) (*Z* = 3.05, *p* = 0.002). Participants spontaneously reported a warmer and more complete sound with S1 (“optimal audibility”) in response to the paired comparisons. The rating of mixed voices (male voice in the background of two female voices) did not differ over the three BCD settings. For traffic noise S3 (“reduced audibility, least distortion”) tended to be significantly higher valued than S1 (“optimal audibility”) (*Z* = 2.1, *p* = 0.03), but after correction for multiple comparisons, this difference was no longer significant. For the own voice, S2 and S3 (“moderate audibility” and “reduced audibility, least distortion”) were rated significantly higher than S1 (“optimal audibility”) (S2: *Z* = 2.9, *p* = 0.004; S3: *Z* = 2.2, *p* = 0.03), indicating that extra low-frequency amplification was not appreciated.

The fundamental frequencies of the own voices varied between 113 and 236 Hz with an average of 165 Hz and the overall intensity varied between 56 and 73 dB SPL with an average of 63 dB SPL. Within-subject intensity differences were small (2 dB) over the different gain settings. No significant correlations between fundamental frequency and setting-rating were found nor were there significant correlations between intensity and settings.

### Speech Intelligibility Tests

**Fig. 6.**
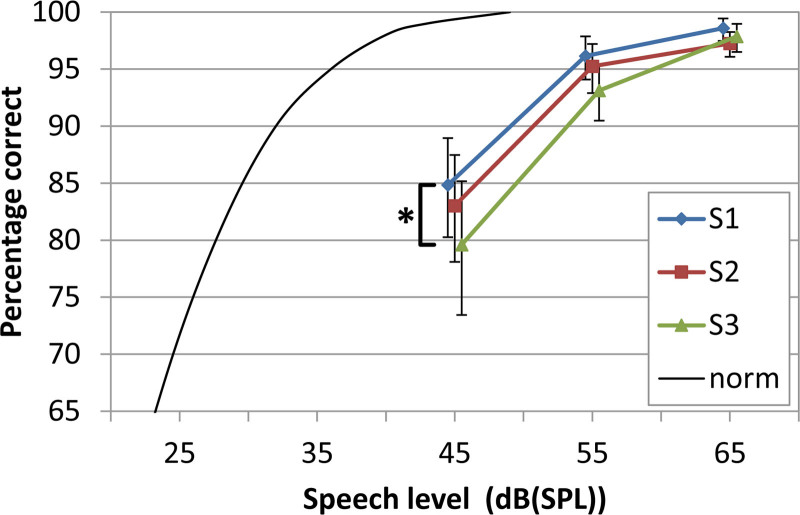
Speech intelligibility in quiet 1 (phoneme scores of CVC words) for the three different adaptation strategies S1, S2, and S3 at different speech levels. Error bars show SEs. The black curve is the norm curve for the CVC phoneme scoring in adults with headphone. *Indicates a significant difference (*p* < 0.05) between S1 and S3 at 45 dB SPL. CVC indicates consonant–vowel–consonant; dB SPL, decibel sound pressure level.

Figure 6 shows the average scores of speech intelligibility in quiet for the three different strategies. The scores ranged from 79% at 45 dB SPL up to 98% at 65 dB SPL. After correction for multiple comparisons, only the speech score of S3 (“reduced audibility, least distortion”) was significantly smaller than the score of S1 (“optimal audibility”) at 45 dB (*t* = 2.81, *p* = 0.008). The phoneme curves were 15 dB (S1) to 18 dB (S3) shifted compared to the norm curve at 45 dB SPL, and the increase in phoneme score with increasing speech level was somewhat smaller than the slope of the norm curve. The speech intelligibility scores at 45 and 55 dB SPL correlated significantly with average BC in-situ thresholds at 1, 2 and 4 kHz (45 dB SPL: *r* = −0.68, *p* < 0.002 for all settings; 55 dB SPL: *r* = −0.58, *p* < 0.011 for all settings; 65 dB SPL: *r* = −0.34, *p* > 0.05 for all settings), but not with the average thresholds at 250 and 500 Hz.

**Fig. 7.**
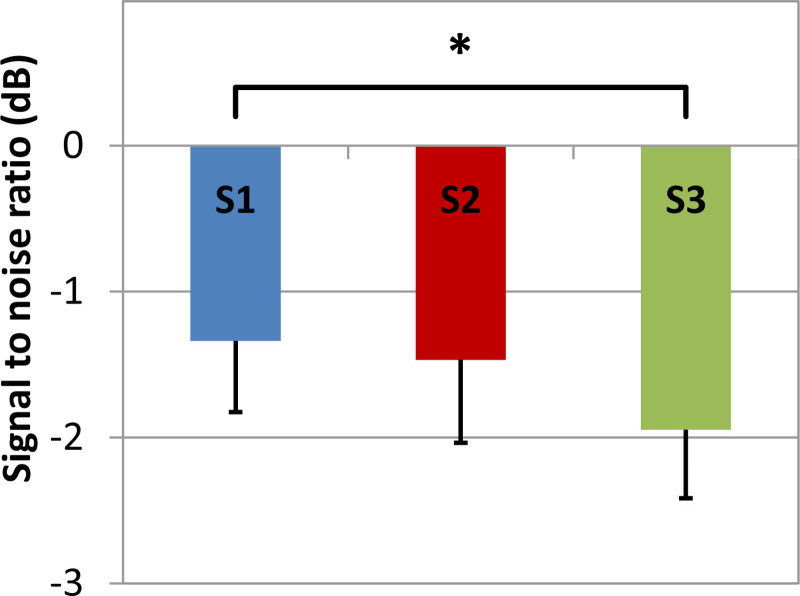
Speech intelligibility in noise (speech reception thresholds) for the three different adaptation strategies S1, S2, and S3 with error bars indicating SEs and *indicating a significant difference (*p* < 0.05) between S1 and S3. Lower values indicate better performance.

Figure 7 presents the SRT for speech in noise with settings S1, S2, and S3, respectively. S3 (“reduced audibility, least distortion”) yielded significantly better speech intelligibility in noise than S1 (“optimal audibility”) (*t(17*) = 4.49, *p* < 0.0003). The SRTs of all conditions were significantly higher than the norm value (S1: (*t(20.4*) = 8.08, *p <* 10^−7^; S2: (*t(19.5*) = 6.81, *p <* 10^−5^; S3: (*t(20.6*) = 7.15, *p <* 10^−6^). The SRTs correlated significantly with average BC in-situ thresholds at 1, 2, and 4 kHz for S1 and S3 (S1: *r* = 0.55, *p* = 0.02; S2: *r* = 0.44, *p* = 0.05; S3: *r* = 0.53, *p* = 0.03), but not with the average thresholds at 250 and 500 Hz. The SRT is more strongly correlated with the average of thresholds of 2 and 4 kHz (S1: *r* = 0.66, *p* = 0.003; S2: *r* = 0.55, *p* = 0.02; S3: *r* = 0.62, *p* = 0.006) (c.f. Smoorenburg 1992).

### Harmonic Distortion

For a fundamental frequency of 250 Hz, the measured harmonic distortion was almost independent of gain settings and input level. The harmonic distortion was within 7.8 to 8.5% for all settings at 65 dB SPL and ranged from 8.9% (S1, “optimal audibility”) to 7.7% (S3, “reduced audibility, least distortion”) at 75 dB SPL input. For a fundamental frequency of 500 Hz, the harmonic distortion ranged from 2.6% (S1) to 1.8% (S3) at 65 dB SPL input, and from 9.2% (S1) to 1.0% (S3) at 75 dB SPL input. For a fundamental frequency of 1000 Hz, the harmonic distortion was below 1% for all conditions.

A further analysis showed that the harmonic distortion at 250 Hz remained in the range of 8 to 12% for all output levels, but at 500 Hz the harmonic distortion increased to 15% at an output level of 110 dB FL.

In a subjective judgment, the authors listened to the output of the BCD with the monitor function of the skull simulator. The sounds of the paired comparisons were used as input. Between settings, differences in timbre were noticeable, but no distortion was audible.

## DISCUSSION

In this study, the NAL-NL2 fitting rule was transformed for application BCDs. Low-frequency output of the BCD is limited (below 1 kHz), so the NAL-NL2 prescribed output cannot be realized, and we had to adopt the prescribed output to suit these low-frequency constraints. Three different adaptations to low-frequency device constraints were compared to find optimal values for amplification and compression. We assessed to what extent perceived sound quality and speech intelligibility in quiet and noise are dependent on the adapted gain for low frequencies.

### Sound Quality

Perceived sound quality depended significantly on the low-frequency gain settings and this dependency varied for the sounds used. S2 (“moderate audibility”) seems to balance audibility and disapproval of a sound with 12 dB head room for loud speech in combination with NAL-NL2 compression settings. It appeared to be the overall best-rated adaptation for sound quality as it had a neutral or positive score for all sounds in the paired comparison experiment. Besides the gain setting, other factors may have influenced the judgment of the sounds, like differences in spectra and the appreciation of the sounds.

The spectra of the sounds were different, which may have influenced perceived sound quality. For the “Mixed voices” stimulus, the energy decreases below 1 kHz. This is an explanation for the finding that no significant differences in rated sound quality between settings were found for this stimulus. On the other hand, the mean spectrum of the own voice shows that most energy is below 1 kHz. For own voice, the highest quality rating was for S2 (“moderate audibility”) and a clear dissatisfaction for S1 (“optimal audibility”) was found. The spectra of “Traffic noise” and “Music” have also clear low-frequency energy contributions and are comparable to each other. Nevertheless, sound quality ratings are different, indicating that spectral differences cannot fully account for the perceived differences in sound quality.

The degree to which a sound is appreciated, irritating or disturbing in its nature, thus apart from gain settings, may also play a role in the relative quality ratings. Although audibility of traffic noise can be important for a person’s safety, the extra low-frequency gain of S1 (“optimal audibility”) was clearly not appreciated, possibly as a consequence of the disturbing, unpleasant nature of the sound. For music, the opposite seems to be true. More low-frequency amplification was appreciated as this resulted in a richer and more pleasant sound.

We also investigated whether harmonic distortion may have had an impact on the perception of the different sounds. There seems to be no specific cutoff point for when harmonic distortion becomes disturbing, but less than 10% is regarded inaudible (Agnew 1998). As the measured harmonic distortion was just below this 10%, it is likely that harmonic distortion has not had a significant influence on the speech tests and the paired comparison of the different settings. This is supported by the fact that in the speech recognition scores no negative effect of harmonic distortion is seen, as the scores of S1 (“optimal audibility”) were not significantly below the scores of S3 (“reduced audibility, least distortion”). Moreover, no distortions were audible in the skull simulator monitoring of the output of the BCD. Only for the own voice, some influence of harmonic distortion might have played a role. The own voices had a dominantly low-frequency spectrum at the position of the BCD (Fig. 4), and this specific intensity distribution may cause the harmonic distortion being heard, as the harmonics may arise above the spectrum. Further analysis is needed but is beyond the scope of this study.

Previous studies (Snik 2015; Hodgetts and Scollie 2017) reported adverse ratings of low-frequency amplification for the patients’ own voice and advised a reduction of the amplification for these frequencies. The dissatisfaction with S1 (“optimal audibility” with most compression and hence most low-frequency amplification) for the own voice found in this study is in line with these results. Patients reported that their own voice sounded hollow or loud. In search of relevant factors, we recorded and analyzed the own voice. We did not find a significant correlation between the fundamental frequency of the participants' own voice and relative quality ratings of gain settings. Therefore the hypothesis that BCD users with a low fundamental frequency perceive better voice quality with less low-frequency amplification than BCD users with relatively high fundamental frequencies must be rejected. In addition, we did not find a significant correlation between the intensity of the own voice and the rating of gain settings. This means that we did not find feedback, that is, speaking in a softer voice to minimize adverse effects of too much amplification if more low-frequency amplification is applied.

It should be noted that S2 (“moderate audibility”) had a higher quality rating for the own voice, than S3 (“reduced audibility, least distortion”). This finding differs from the large low-frequency reduction applied in the DSL-BC and is an indication that the reduction in the DSL-BC prescription may be too large (Hodgetts and Scollie 2017).

### Speech Intelligibility in Quiet and Noise

Speech intelligibility in quiet was hardly dependent on low-frequency gain. From Figure 3, we observed that the gain adjustments caused the largest reduction of audibility at 250 Hz. For frequencies below 500 Hz, the contribution to speech recognition is relatively small, according to the band weightings used in the SII standard (ANSI 1997). This may explain why the effect of the different settings on speech intelligibility in quiet was negligible for most conditions. Only for soft speech of 45 dB SPL a small decrease in speech score was found for the largest reduction of audibility in adaptation S3 (“reduced audibility, least distortion”). This is an indication that the reduction of output at low frequencies should not be too large.

The average speech scores at 65 and 55 dB SPL were slightly below 100% (Fig. 6). This was due to one patient with maximum scores that varied between 64 and 79% over the settings and several participants with elevated thresholds at 2 and 4 kHz.

For speech intelligibility in noise, a significant better SRT was found for S3 (“reduced audibility, least distortion”) compared to S1 (“optimal audibility”), but the difference was only 0.6 dB. This difference corresponds to about 9% speech intelligibility, which is only a small improvement. This improvement is most likely due to less upward spread of masking in S3 compared to S1.

The average SRTs in noise were significantly worse than the norm value in all settings. Most likely this is mainly due to the sensorineural part of the hearing loss. Smoorenburg (1992) investigated the relationship between pure tone threshold and SRTs in noise-induced hearing loss. He reported a formula to relate the average decrease in SRTs to the thresholds at 2 and 4 kHz. We applied this formula to our data and found a predicted SRT loss of 3.9 dB. Given the norm value of −5.5 dB, the predicted SRT is −1.6 dB. This corresponds very well with the average SRTs found in this study.

### NAL-NL2 Prescriptive Targets for Bone Conduction Devices

Application of the NAL-NL2-BC prescription resulted in sufficient speech intelligibility in quiet at normal and confident speech levels. The effective hearing loss after application of the NAL-NL2-BC gain can be derived from the shift of the 50% point of the phoneme curves compared to the norm curve, as this shift highly correlates with the pure tone average of 500, 1000, and 2000 Hz (Guidelines for determining threshold level for speech 1988). This shift is estimated to be 15 to 18 dB for all settings. This value is comparable to the mean BC in-situ pure tone average of 500, 1000, and 2000 Hz of 21 dB, which means that, on average, the air-bone gap is effectively bypassed. One would expect more effective gain, compensating for part of the sensorineural hearing loss. However feedback prohibited fitting high frequencies to target for some patients and below 1 kHz the fitting is below targets due to the device constraints.

The average SRT in noise can be explained by the BC thresholds in the higher frequencies, as argued in the previous paragraph. This suggests that the influence of reduced audibility on the SRT is absent or small, which is an indication that the NAL-NL2-BC prescribes sufficient output.

As discussed in the previous paragraphs, it is possible to apply a reduced low-frequency gain to adapt the NAL-NL2 prescription to device constraints without considerable negative effects on speech intelligibility in quiet and noise. Together, these findings show that the adapted NAL-NL2 prescription is suitable for application in BCD fitting. However, study participants only experienced the different programs for 1 to 2 hours in a laboratory setting and the acclimatization with approximately 1 hour only was short. Furthermore, the amplification of the BCDs of patients before our study was not uniform. Therefore, the results of this study need to be confirmed in a study that focuses on the real-life experiences and performance with the NAL-NL2 prescription after sufficient acclimatization to the new settings.

This study focused on low-frequency device constraints, but for elevated BC hearing thresholds in the higher frequencies, the NAL-NL2-BC prescription may be also limited by other device constraints. The maximum stable gain, obtained with a feedback analysis, may limit the gain and the MFO may limit the output. The effect of these limits should be studied to define the fitting range of a BCD for optimal NAL-NL2-BC–based fitting.

To obtain a better evidence base for the NAL-NL2-BC prescription rule, the effect of the different NAL parameters (experience, gender, tonality, bi/monaural) should also be studied. Participants in this study were experienced BCD users, but new BCD users may perceive the prescribed output as too loud, because these users may be used to less sound, due to their hearing loss. And even the experienced users of this study may have had quite a different sound experience from what they were used to. The gender difference (women prefer less gain than men; Keidser et al. 2011) is present in AC hearing aids and may be related to factors in the pathway of the sound. It is currently unknown whether a gender difference also exists in BCD fitting. For tonal languages, the gain prescribed by NAL-NL2 for the lower frequencies is greater in the lower frequencies, compared to the gain for non-tonal languages (Keidser et al. 2011). Therefore, the adaptations to the low-frequency gain used in this study may have other effects for tonal languages, although these effects may be relatively small.

### Limitations

This study has some limitations. First, we only used one specific BCD with its own device specificities such as resonance frequency or distortion, although the effect of the latter is thought to be limited as already discussed. Second, a limited number of sounds was used for a fairly short time. Different sounds and a longer acclimatization period will increase the evidence for a specific gain setting. Third, cochlear losses of the participants were limited. For larger cochlear losses, the available dynamic range is further reduced and one would expect extra value of amplification (Gawliczek et al. 2020).

## CONCLUSIONS

With a moderate gain adaptation in the low frequencies due to device constraints the transformed NAL-NL2 prescription is suitable for fitting BCDs. It resulted in a neutral or positive relative sound quality for all stimuli without negative effects on speech intelligibility. A moderate gain adaption below 1 kHz seems to balance audibility and sound quality for speech intelligibility and perceived sound quality of different sounds in BCD users. The NAL-NL2 BC prescribed a sufficient amount of gain to effectively bypass the air-bone gap, as indicated by the speech tests. Further research is needed to extend these findings to long-term use and real-life experience.

## ACKNOWLEDGMENTS

The authors gratefully acknowledge the participants and they thank Marloes Adank for her help with data collection, Serge Kriek, Tove Rosenbom, Simon Krogholt, Patrick Maas, and Jan Petersen from Oticon Medical for their input in setting up the protocol and EMID for an extra SKS 10 during the study.
